# Guy's Hospital Reports

**Published:** 1838-07

**Authors:** 


### Art. III.

*Guy's Hospital Reports.* No. V. *October*, 1837. Edited by G. H. Barlow, m.a. and l.m., and J. P. Babington, m.a. &c.—*London.* 8vo. pp. 260.

The present Number of the Guy's Hospital Reports does no discredit to its predecessors. The papers by Dr. Bright and by Mr. Taylor, in this, as well as in some of the former Numbers, take the lead; but are accompanied by several others of very considerable value. The highly practical character of almost all of them is precisely that which should distinguish a book of the kind. Authentic and well-digested histories of individual cases of disease, unembarrassed by superfluous theory and vague speculation, tend in a much higher degree to the diffusion of useful knowledge among the profession at large than many of the more ambitious "systems" and elaborate dissertations of a former day. With very much to commend in the Number before us, we have still, however, to regret that more industry is not exhibited, in some of the papers, in the work of condensation. The number of medical periodical works which are now published throughout Europe, all claiming a share of public notice, is so great, that any one in which this indispensable qualification is overlooked cannot safely reckon on an extensive and permanent perusal. We shall notice all the papers that are of most importance as fully as our limits will permit.

1. *Observations on the Ganglionic Enlargement of the Pneumo-gastric Nerve; the probable Function of that Ganglion; and the Position which it occupies in the Human Subject, and in several of the lower Animals.* By Mr. Edward Cock.

The object of this paper is to show that the pneumo-gastric nerve resembles somewhat, in structure and function, one of the fifth pair of nerves, or a spinal nerve. It contains, like them, two sets of filaments,—the one for sensation, the other for voluntary motion; and to these is added a third set, imparting to it its peculiar character as a respiratory nerve. The nervous fibrillæ composing the pneumo-gastric are found, at their origin from the medulla oblongata, to be in apposition with three sources of nervous power: the corpora restiformia, or common sensory columns of the spinal cord; those fibres of the corpora pyramidalia, or anterior motor columns, which Mr. Solly has described as passing into the cerebellum; and the olivary bodies. From the first of these origins proceed the filaments for common sensation, and for "affording to the lungs, pharynx, œsophagus, and stomach that faint but peculiar sensibility which they appear to possess; filaments which impart to the stomach the sensation of fulness when that organ has been distended with food; to the lungs the 'besoin de respirer,' and the sensory functions necessary for respiration." From the second origin arise those filaments which communicate the power of motion, as exemplified in the laryngeal muscles; and from the third the portion of the nerve "constituting its specific character." The ganglionic enlargement of the pneumo-gastric nerve, which, in the human subject, is situated just beneath the foramen lacerum, is considered by Mr. Cock to be analogous to the ganglion on the posterior root of a spinal nerve; and, by dissection, he has been enabled to ascertain, in the human subject, and much more distinctly in the sheep and some other animals, that the pneumo-gastric trunk, as it leaves the base of the skull, consists of "two orders of filaments,—viz. the ganglionic and the ganglionless. The former terminate in the ganglion, where their fibrous character becomes lost, after the manner of the posterior roots of the spinal nerves; the latter are continued downwards beyond the ganglion, having merely a cellular connexion with it, and resembling in this respect the motor portion of the fifth pair. Lastly, from the ganglion arise two sets of nerves: the one constitute the laryngeal; the other join the ganglionless filaments mentioned above, and form part of the trunk of the par vagum, descending to the chest." The superior laryngeal nerve arises from the ganglion, and appears to derive some very minute fibrillæ from the pneumo-gastric trunk above the ganglion, which may be either "specific respiratory filaments or motor-muscular filaments; perhaps both." It is distributed, with the exception of a small muscular branch, and of two branches which communicate with the inferior laryngeal, exclusively to the mucous membrane of the larynx, and is supposed by Mr. Cock to communicate to that organ its exquisite sensibility. The inferior laryngeal, or recurrent nerve, which is distributed to the muscles of the larynx, is presumed to be derived from the motor root.

The ganglion of the pneumo-gastric nerve is called by the author the "superior laryngeal ganglion," as being essentially connected with the superior laryngeal nerve: its situation varies considerably in different animals, being in some nearer to, and in others farther from, the base of the skull; but in all it corresponds to the point at which the superior laryngeal nerve is given off from the pneumo-gastric trunk, that nerve being uniformly derived from the ganglion. The appellation just mentioned was first applied to the ganglion by Sir Astley Cooper, who first suggested to Mr. Cock the views which are detailed in his valuable paper.

### II. *On the Distribution and probable Function of the Laryngeal Nerves.* By Mr. John Hilton.

This paper forms an appropriate adjunct to the one which we have just considered, as it contains an account of the distribution of the superior and inferior laryngeal nerves, the sensory and motor nerves of the organ of voice. It is not necessary to enter into the details which it presents, as the facts disclosed, and the conclusions to be drawn from them, may be summed up in a few words. It appears, from Mr. Hilton's dissections, 1st, that the superior laryngeal nerve, with the exception of a branch to the crico-thyroid muscle, and two filaments which communicate with the recurrent nerve, is distributed exclusively to the mucous membrane, cellular tissue, and glands; from which it follows that this nerve is a nerve of sensation: 2dly, that the inferior laryngeal or recurrent nerve alone supplies all the muscles which act immediately upon the column of air passing to and from the lungs, and is therefore to be regarded as the motor nerve of the larynx: conclusions which accord with the views contained in Mr. Cock's paper.

III. *Observations and Experiments on the Lungs of New-born Children, in relation to Medical Jurisprudence.* By Mr. Alfred Taylor.

The discrepancy of opinion which still exists amongst the best medical authorities, as to the utility of the hydrostatic test in suspected cases of infanticide, induced Mr. Taylor to put it to the proof afresh on the lungs of four infants, the circumstances connected with whose birth were known. His systematic mode of investigating these cases presents a good example of the manner in which such examinations should be conducted. The questions which he endeavours to solve experimentally, and altogether independently of his previous knowledge of the facts, are the following:

1st. *What was the age or degree of maturity of the child?* The elements for the determination of this point are—the length of the body; insertion of the umbilical cord, whether in the centre of the length of the body; the weight; quantity and length of hair on the head; firmness of the bones of the cranium, facility of their overlapping on compression; development of the nails; descent of the testes; presence or absence of membrana pupillaris; state of the skin,—whether desquamating, reddened, or discoloured in any part.

2d. *Had the child lived to respire?* For the resolution of this question the condition of the body must be noted: whether full and plump, or the reverse; the development of the chest; the size and colour of the thymus gland; the prominence of the heart; the degree in which the lungs advance over the pericardium,—their colour, whether livid or of a bright red, and whether crepitant on firm compression; the condition of the ductus arteriosus and of the foramen ovale; the weight of the lungs as compared with that of the whole body, (Ploucquet's test,) and the hydrostatic test,—i. e. their specific gravity. Before investigating this latter point, we must satisfy ourselves that putrefaction has not commenced.

After separating the lungs from the heart and thymus gland, we are next to observe whether each of them float equally well. They are then to be enclosed in a cloth and firmly compressed, in order to ascertain whether the air can, as in cases of artificial respiration, be partially expelled from them. One of the lungs is next cut into ten or twelve pieces; the colour of the sections, the crepitation (if any), and freedom from disease, are noted. If these portions likewise float, compression is to be repeated on each of them, (taking care not to carry it so far as to destroy their organic texture,) and we again try their buoyancy.

One of the lungs of the first subject examined by Mr. Taylor was inflated to the greatest extent, (to a degree, in short, which would have been impracticable whilst within the chest;) when it was found to become universally of a bright pink-red colour, not distinguishable (though the child was born dead) from that of lungs which had respired; and it was strongly crepitant.

Extravasation of air beneath the surface of the enveloping membrane is a frequent, but by no means invariable, occurrence in artificially dilated lungs. In the case just alluded to, the air could not be expelled from the lung whilst entire, in such a degree as to cause its sinking; but on being cut into pieces, and on these being similarly treated, they fell to the bottom. "When the lungs have been fully distended with air by respiration, on the contrary, it is impossible to force out that air by the mechanical compression of any of the divided portions." Compression of the lung was long since proposed by Béclard, and also by some of the German medical jurists, as furnishing a diagnostic sign of artificial dilatation. Mr. Jennings and Mr. Taylor, about five years ago, and almost simultaneously, brought forward this test in this country; each being unconscious, at the time, of the other's investigations on this head, as well as of the previous ones of their continental fellow-labourers.

Very forcible inflation of the lungs of living animals has a fatal effect, as was shown many years ago by M. Leroy d'Etiolles; and Martini enumerates this amongst the modes of perpetrating infanticide: but the production of such a result implies a very great degree of violence in the operation, and such as we can hardly suppose would ever be employed where the object was legitimate,—namely, to resuscitate the child. Now it is only when the process has been carried to this extreme length that there will be any great difficulty in expressing the air from the artificially dilated lung; and, consequently, Béclard's test remains of considerable practical value. When animals have been killed by violent insufflation of the lungs, if death be immediate, the air-cells are not found lacerated; but, in those which live for some minutes, air is extravasated into the chest. The full inflation of the lungs by artificial means is not so easily effected as is commonly thought; and the more immature the child, the more difficult is it, as Béclard has remarked.

3d. *Was the child born alive?* To answer this query independently of the state of the lungs,—for a child might be born alive, and put to death before it had yet made a first inspiration,—we must examine the condition of the cord; the changes occurring in which, as diagnostic of life, are laid down at length by M. Billard, Devergie, and others: yet, as Mr. T. well remarks, these changes are often not by any means well marked, nor much to be trusted to even when they do exist, if respiration has not taken place; and, where it has, they become of very secondary importance.

In his first case, though the child was born dead, yet there was, at the junction of the sound with the shrivelled skin, a faint line of redness, like that of incipient inflammation, gradually diffusing itself towards the skin of the abdomen; and the only difference between the cord in this instance, and another where the child had survived twenty-four hours, was in the dryness and transparency of the latter. Our author is altogether sceptical as to the possibility of telling, from the appearance of this part, the exact period of survival.

A diffused redness observed about the neck and shoulders in the first case detailed, was admitted as sufficient proof of the child having been alive about the period of birth. The pulmonary vessels were in the same case distended with blood, so that those jurists are in error who look upon this as a proof of respiration having commenced.

"The mean weight of healthy lungs, calculated from their weight in six mature children which had died without breathing, I found," says Mr. Taylor, "to be 569 grains; while the weight of well-developed organs, which have fully respired, is rarely under 1000 grains." Respiration differs from all other causes which render the lungs buoyant in water, in the fact that this process increases their absolute weight, while it diminishes their specific gravity. Of all the physical changes in the lungs as evidence of respiration, the least liable to fallacy is that connected with the increase of weight. The lungs may be of a light red colour, fully prominent in the chest, highly crepitant to the feel, and very buoyant in water; yet, if their absolute weight be not raised above that of the fœtal condition, it is almost, if not absolutely, certain that respiration has not been performed.

"Ploucquet's test," says Mr. Taylor, "although very far from being infallible, is capable of serving as a good corroborative proof." The mean weight of the lungs, in three children which had breathed, was found to be to that of the bodies as 1 to 35: thus coinciding exactly with Ploucquet's results. But the ratio in regard to those which had not breathed (deduced from four cases of mature infants) was as 1 to 55; and not as 1 to 70, as laid down by the German author, who seems to have formed his conclusion from inadequate premises; namely, from two children born dead and an immature one which had breathed. M. Devergie has found the test inapplicable to children which have not reached the eighth month of gestation. In one which had lived twenty-four hours, the ratio was only 1 : 41.

Mr. Taylor has never known lungs artificially inflated to be of an uniform light red colour throughout, except when the process has been carried to its very fullest extent; and then there is commonly distinct crepitation. An uniform light red colour, without crepitation, he looks upon as characteristic of lungs which have respired.

When respiration has been imperfect, Mr. T.'s experiments induce him to believe, contrary to some authorities, that, by compression, the air may be so far expelled from the divided portions as to cause them to sink in water. In such cases of feeble respiration, the air has probably passed only into the larger divisions of the bronchi, and not into the minute ramifications and the air-cells. Notwithstanding all this, compression must still be considered a valuable adjunct to other tests: it will, at least, distinguish all cases of complete respiration from those of artificial inflation. "It must, I think," he continues, "be admitted that there are no means of distinguishing feeble respiration from artificial inflation which has been carried only to a slight extent. In a case of this kind, I apprehend, the only course left open to a medical witness, is to state to the jury that the evidence derived from experiments on the lungs left it uncertain whether the child in question had respired or not. The jury will then know how to return their verdict; for it must be remembered that they have always circumstances to guide their judgment as well as medical opinions; and it is upon the whole, and not upon a part, of the evidence laid before them that their verdict is founded." In point of fact, the plea of artificial respiration can rarely be put in with any degree of plausibility in defence of the prisoner. Mr. T. knows of but one case on record (in Bohn, and cited by Meckel,) in which the mother artificially inflated the lungs. Hence the hydrostatic test retains a high degree of practical value; and, in the rare instances where it fails, its deficiency can, at the worst, only lead to the acquittal of the guilty, and never to the condemnation of the innocent.

The invisibility of the air-cells is mentioned by most writers as a proof of the air in the lungs having been derived from respiration; whilst, where they are large and obvious to the naked eye, if decomposition has not commenced, extravasation from violent inflation has been generally presumed. This, however, is not an infallible criterion, as, in one of the cases in the present paper, " the air, although undoubtedly derived from respiration, was scattered over the whole surface of the lungs in large visible vesicles."

The absurdity of the English law of infanticide, according to which the jury cannot find a verdict of murder, provided the child, though wilfully put to death, and that too, perhaps, after it has fully breathed, was not yet altogether separated fr om the mother, is commented on with merited severity.

The second case detailed exemplifies the possibility of a child living and breathing for so long a time as half an hour after birth, without there being any evidence, even after a careful examination of the lungs, ductus arteriosus, foramen ovale, &c., to show whether it was born alive or dead.

The third case is likewise an interesting one, as the lungs sank in water, though life had been prolonged for six hours. The greater part of this time, however, the child was in strong convulsions, during the continuance of which it made violent efforts at inspiration. The lungs were crepitant and obviously contained air, and the cells on some parts of the surface were quite visible to the unassisted eye. After incisions had been made, so as to allow some of the venous blood with which these organs were gorged gradually to escape under water, they floated: on dividing them into portions, and compressing them anew, they again sunk. The hydrostatic test as well as Ploucquet's, and the colour and volume of the lungs, would all in vain be appealed to in such a case to decide whether the child had breathed. The ductus arteriosus and foramen ovale, moreover, were still unchanged. In short, by a rash observer, the case would most readily have been mistaken for one in which artificial respiration had been practised. Instances like this prove the absurdity of concluding positively, as is so often done, that the child was dead-born because the lungs sank in water. Desiccation and transparency of the chord are considered by Mr. T. to afford better evidence of the child having for some time survived its birth than the line of redness occasionally observed at the point of future separation. In the case just alluded to, it was found flattened, horny, and transparent; the umbilical vein being contracted and dried up, as it were, to a filament of coagulated blood.

The fourth case likewise is somewhat remarkable, inasmuch as the lungs, and even small divided portions of them, sunk in water, though the infant had survived its birth for twenty-four hours. Such exceptional cases, however, are less rare than some have supposed. Similar ones have been recorded, as Mr. T. remarks, by Bernt, Remer, Orfila, and others. Most of such instances have been furnished by premature children, but by no means all. The recent researches of Dr. Joerg, of Leipzig, are thought by Mr. Taylor to throw some light on the cause of these obscure cases. He conceives that the act of parturition, and especially its duration, seems to prepare the system of the child for the efforts it has to make in inspiration. If parturition be too rapid, the child may be born before it is yet endowed with the organic stimulus necessary to induce the first act of inspiration. " A parturition of the natural duration (on the contrary) gradually checks the placental circulation, and limits that of the fœtus chiefly to its own system; while it engenders in the latter a gradually increasing and finally an urgent want of some new mode of inspiration. Supposing the first inspirations to be, from any cause, feeble or imperfect, then the organs will become only partially distended: the remaining portions will preserve their fœtal condition. Dr. Joerg considers this as a positively diseased state of the lungs in the new-born child, and he has given to it the name of *atelectasis.* Into its supposed causes we have not room to enter. Dr. J. has remarked that those portions of the lung which are not speedily distended by air afterwards become consolidated or hepatized, so that all traces of their vesicular structure are lost. The length of time which the child survives will depend upon the degree to which its lungs have become dilated. Mr. Taylor alludes to a case which he met with himself, in which the child survived for six months with a large portion of the lung in this unexpanded or fœtal condition.

To conclude: though the pulmonary tests do sometimes fail us in our endeavours to prove live-birth, yet such cases are merely exceptional. In a very great majority of instances, the signs thus furnished are so decisive as to leave us nothing to wish for. Thus, if "in the body of a healthy full-grown child, which has but recently died, we find the lungs filling out the cavity of the chest, of a light red colour, spongy and crepitant beneath the finger, weighing at least two ounces, and, when divided into numerous pieces, each piece floating in water, even after violent compression, is it possible," asks our author, "to doubt that respiration has been performed?"

To discard such tests altogether, as some would have us, merely because they are not in every case infallible, would be just as wise as to disregard the ordinary chemical and pathological proofs of poisoning because these are found in some instances unsatisfactory. We need hardly add, after this analysis of Mr. Taylor's paper, that it is most valuable, and highly deserving the attention of every medical jurist.

### IV. *Cases by* Mr. Bransby Cooper.

*Gangrene of the Hand.* A carpenter, aged eighteen, of delicate constitution, struck the end of the nail of one of his fingers against a piece of lead: there was only a slight ecchymosis under and above the nail. On the third day, a numbness was felt in the finger adjoining the injured one; and, before a fortnight, all the fingers of that hand were in the same state—numb and bloodless, but painful; the pain complained of being principally referred to the ends of the fingers. During all this time he suffered pain in the course of the median and external cutaneous nerves, but the general health remained unaltered. On admission (October 7,) three weeks after the accident, he was very weak; his hands and feet cold. The pulsation of the brachial artery, high up, was cord-like; at the bend of the elbow, it was undistinguishable; and, in the centre of the fore-arm, it could be felt again in the radial, but not in the ulnar artery: in several places the artery was tender to the touch. A rough, sawing noise could be heard by the stethoscope in the subclavian artery, at a spot where the vessel was pressed forward by a small tumour, probably an enlarged lymphatic gland. The fingers and thumb were livid, cold, and insensible; the end of the injured finger in the condition of dry gangrene: no attempt, as yet, at a line of separation. During a week he had bad rest; quick, small pulse; headaches; sickness of stomach, and even hiccup: the hand and fore-arm swollen, pitting on pressure, painful, and marked with blue lines in the course of the veins. On the 20th, the line of separation commenced on the dorsum of the hand above the roots of the fingers. From this time both the local and constitutional symptoms began to abate. On the 31st, the line of separation had gone through all but the tendons and bones; and, on the 11th November, the hand was removed by Mr. Cooper at the radio-carpal articulation. Henceforward the patient rapidly convalesced; the gland in the axilla disappeared, and the pulsation in the subclavian artery became much more natural. January 6th, discharged cured. It is not stated whether the brachial and ulnar arteries regained their pulsation after the cure.

The recovery of this patient leaves us nothing but speculation as to the cause of the gangrene. Can we believe, with Mr. Cooper, that the mechanical pressure of the enlarged gland on the axillary artery could have produced such an effect? Scarcely. Even he himself regarded this enlargement as secondary to the gangrenous inflammation. To say that it may have been the consequence of "a general constitutional derangement of the vascular system," is only to apply a vague expression, which we cannot well understand, to the explanation of an incontrovertible fact. Or, to suppose that the trifling injury sustained in this case, even though accruing to a limb while under the influence of both these assumed conditions produced it, is bringing us little nearer to the mark. What then was the cause? Mr. Cooper alludes to the possibility of the presence of *arteritis*, but at the same time gives his objections against such, as the cause of the gangrene: objections, however, in the validity of which we do not fully concur; for we have seen a case of *spontaneous* gangrene of the hand taking a course very analogous to that related by Mr. Cooper, in which intense inflammation of the axillary and brachial arteries was the real and only cause, though scarcely suspected during life. We are, therefore, inclined to consider the gangrene in question as the consequence of arteritis.

*Popliteal Aneurism ;—Relapse after Operation.* An Italian gentleman, aged thirty, consulted Mr. Cooper for an aneurism in the left popliteal space. Eight months previously, he had been operated on by a surgeon in Manchester for the same disease. The pulsation in the tumour had, on that occasion, ceased as soon as the ligature was applied, but returned in twelve hours after, and has continued slowly increasing ever since. Mr. Cooper tied the vessel close above the tumour. "Immediately the ligature was applied to the artery, the pulsation in the tumour ceased, but in a few minutes, became again as distinct as before the operation:" however, "the patient had entirely lost the painful sensation in the limb which existed prior to the operation." Considerable febrile reaction followed, with pain, tension, and throbbing in the wound and tumour. On the third day, the pulsation in the tumour again ceased, and the sac felt empty, as if no coagula had existed in it. Up to the eighth day, some fluid blood could be felt in the sac, though admitting of being squeezed out by pressure. On the twenty-second the ligature came away. An inordinate action of the heart and arteries, which, from the time of the operation, disturbed the patient, and gave rise to apprehensions of internal aneurism, subsided completely by a fortnight's residence at the seaside.

Why did the first operation in this case fail? Without supposing that there was any error committed in its performance, (for there is every reason to believe that *an* artery was tied,) many explanations of the cause of the failure may be offered. In the *first* place, there may have been a double femoral artery with reunion of the branches above the aneurismal sac, as noticed by Sir C. Bell, in Anderson's Quarterly Journal; and by Dr. Houston, in the Dublin Hospital Reports: this is the most probable explanation. *Secondly*, the return of the pulsation may have arisen from an enlargement of the anastomoses connecting the profunda with the anastomotica magna; though the earliness of its reappearance can scarcely be accounted for in this way. *Thirdly*, a branch arising from the femoral, high up, and either rejoining that vessel or opening directly into the sac itself, as observed by Sir A. Cooper and Scarpa, might have perpetuated the pulsation.

As regards the temporary recurrence of pulsation, after the second ligature, it may be accounted for on the very probable supposition that an enlargement of the anastomosing branches had been brought about by the first attempt, sufficient to carry, for a time, a small quantity of fluid blood into the aneurismal sac. The rapid subsidence of the pulsation almost justifies the inference that none but collateral vessels were engaged in its production.

*Two Cases of Hernia.* Mr. B. Cooper is an advocate for relieving the stricture without opening the sac. In one of two cases which he relates the practice succeeded well; in the other it comparatively failed. The first patient had no secondary bad symptoms; the second was near dying. Mr. C. states that, in five cases on which he operated in this way, "his patients suffered no more after the operation than they usually do after the successful application of the taxis, while, on the contrary, if the hernial sac be opened, and the peritoneal cavity necessarily exposed, a high degree of constitutional derangement, and subsequent prostration of power, almost always followed." It is but right to add, that Mr. Cooper and his colleague, Mr. Key, differ in opinion on this point of practice. Mr. C. considers that, in the first of the cases reported, the patient obtained immediate and permanent relief, *because* the sac was not opened; and that, in the second, dangerous symptoms followed, *because* it was opened. But, in coming to such a conclusion, the relative positions of the two patients should be fully weighed; as the great secret of success in such cases lies in early operation. Now, it appears, that, in the cases related by Mr. Cooper, the operation in the first was performed on the second day after the occurrence of the strangulation; whilst, in the second, it was delayed to the fifth day. We do not attempt to disapprove of Mr. Cooper's method, but nevertheless we are not satisfied that these two cases, with such differences between them, can settle the point at issue. Regarding the five other cases alluded to by Mr. Cooper, we should have presented to us five of precisely a similar kind treated in the usual way, before we can take them in evidence of the superiority of his peculiar mode of practice. There is every reason for believing that the plan is good: all we ask is, a comparative demonstration of its utility.

*Ununited Fracture of the Humerus; cured.* A healthy young woman with ununited fracture of the humerus, of six months' standing, was admitted into Guy's Hospital. Mr. Cooper, judging from her report that the ordinary means of cure had been used properly, though without success, produced friction of the ends of the bones on each other for ten days; but no union followed. He then passed a seton through the new joint: "the seton was worn ten weeks, but the irritation which, for the first few days held out a prospect of success, soon subsided; and proved ultimately to have led to no happier result than that of the former attempts." A yolk-of-egg bandage was then applied, and persisted in for six weeks, but equally without success; and, as a last resource, an envelopment of plaster of Paris was had recourse to. About the fourth month after admission, when there was no attempt at union, Mr. Colles, of Dublin, who happened to pay a visit to the hospital, suggested the use of mercury. Hydrargyrum c. creta was administered, (four grains, three times a day.) Ptyalism supervened in four days. "In a month, perfect union of the bone had taken place, affording satisfactory proof that the mercury had produced an altered action of the capillaries of the affected part, and exemplifying the alterative influence of that metal." About five months afterwards the same arm was broken, below the seat of the original fracture, and again united in the usual time and under ordinary treatment.

*Wound of the Tongue; fatal.* A tobacco-pipe in a young man's mouth, being accidentally struck in the bowl by a comrade, penetrated and wounded the tongue and pharynx. It was not considered at the time that any part of the pipe, though broken, remained in the mouth. On the sixth day, he discharged apparently from the stomach, about a pint of grumous blood. This discharge recurred repeatedly from the eighth to the twelfth day. The patient became extremely reduced, "but no wound could be discovered to point out from whence the hemorrhage proceeded." He sank fast from repeated discharges of coagula from the stomach, "which reduced him to so low a state that transfusion was contemplated." He died on the fifteenth day, almost immediately after throwing up coagulated blood. On examination, a cicatrix appeared on each side of the tongue, and "a small irregular opening was discovered behind and below the left tonsil." Two inches and a half of the end of the pipe was found imbedded in the substance of the tongue. Blood was found in the stomach and bronchial tubes. The source of the hemorrhage could not be ascertained; but there can be little doubt that the blood had flowed from the laceration in the pharynx, (and not from the wounds of the tongue, which were cicatrized,) and had thence trickled down into the œsophagus and trachea. Mr. Cooper hints that, had the source of the hemorrhage been known during life, a ligature on the carotid might have saved the patient.—Nothing more likely.

*Stone in the Bladder.* Mr. Cooper extracted a calculus from a child, seven years old. The calculus was oval-shaped, and about an inch long. The outer layer consisted of bright, transparent crystals of oxalate of lime, of an octohedral form, with rectangular base; the next, or subjacent layer, of oxalate of lime, with a minute proportion of phosphate of lime; and the third, of carbonate and phosphate of lime, with lithic acid. The deeper layers and nucleus were not examined.

Every one of Mr. Cooper's cases contains some useful practical information; but, for our own sake, and for that of every other reader, we beg that, in future, his cases may be more condensed: few men can wade through three or four reports a day of any disease. This is, however, no farther a fault of Mr. Cooper, and of others whom we might name, than that they are content to print the notes of their pupils instead of their own. Far be it from us to discourage the practice of note-taking by pupils: on the contrary, we believe it to be the best of all exercises in which they can be engaged. All we require is, that medical men, in giving to the world the results of their experience, shall give their own thoughts in their own language. Proxies, in such cases, are apt to be injudicious by their prolixity.

Although not strictly in order, we will here notice a case by Mr. Key, related in another part of the volume.

*Sphacelated Intestine and Omentum in Femoral Hernia.* A lady, aged forty-four, consulted Mr. Key for a femoral hernia. He found it to consist of a small portion of omentum, irreducible from adhesion to the sac. A hollow pad with a truss relieved, for a time, the apprehension and uneasiness. Several months after, the tumour became suddenly enlarged and painful. The taxis failed in giving relief, and the patient would not consent to an operation until five days had elapsed; when the abdomen was hard and tender, the pulse 100, the matters vomited fæcal, and the coverings of the tumour inflamed. As soon as the sac was punctured, a dirty brownish fluid oozed out, and a darkened mass of omentum, in an advanced state of decomposition, was discovered. Beneath this lay a knuckle of intestine, with a patch of sphacelus about one inch in diameter, but without any line of demarcation between the dead and living parts. Having divided the stricture, Mr. Key pressed the intestine gently back towards the abdomen, leaving the major part external to the neck of the sac. The omentum, too, was left in the sac, and the whole covered with a poultice. Little amendment followed for some time. On the fifth day from the operation, a discharge of fæces took place from the wound; on the seventh, the sloughs had cleared away; on the seventeenth, a few hard balls of feculent matter passed, for the first time, from the rectum. In six weeks the wound was completely closed over, and the fæces had taken their natural course. The cicatrix opened slightly on two occasions, but it soon became permanently closed. "The patient continues well to the present time, wearing a truss as a matter of precaution."

In the individual in question, Mr. Key considered that the vomiting had so far emptied the stomach and intestines as to remove the necessity for any further abstraction of their contents by incision. He apprehended that a state of collapse, such as he had often seen follow the free evacuation of the canal by a proceeding of this kind, might have changed his patient's present elastic state to one of depression; and he judged that nature would work her object with more security if allowed a respite after the division of the stricture, than if disturbed by the operation of incision into the mortified intestine. As regards the treatment of the mortified omentum, many surgeons adopt the same practice as that recommended by Mr. Key; but the plan of leaving the deadened portion of the bowel to empty itself by the separation of the sloughs differs from that usually adopted. How far such a plan is to be acted upon as a general rule remains yet to be determined.

### V. *On the Diagnosis of Organic Diseases of the Uterus.* By Dr. Ashwell.

The avowed object of Dr. Ashwell's paper is to render more easy the study of this complicated portion of pathology. He does not profess to put forward much that is new; his aim being classification, not discovery. The methods of enquiry he arranges as follows:—the history of the symptoms; examination by touch; the application of the speculum; the stethoscope; and the investigation of the discharges. In regard to the value of pain as a symptom, he justly observes that, "in chronic structural disease, there is generally little acute, early, or continued pain; while in functional disorders, such as irritation and inflammation, these are invariable conditions." The duration of pain, as also the existence or not of emaciation, as signs in themselves of organic disease, he considers, though essential to be attended to, yet incomplete without the addition of what he states to be the most valuable means of diagnosis,—viz. examination by touch, more especially when aided by the speculum and stethoscope. The directions for conducting the examination by touch are good: he dwells at some length on its efficacy as a means of diagnosis in cases of organic diseases complicated with pregnancy or doubtful pregnancy, but does not appear to us to attach a sufficient degree of importance to the aid afforded by the stethoscope in these investigations. The uterus being liable to be distended or enlarged from various causes, much stress is laid on the vaginal examination. We quite agree with the author in his remarks on the peculiarities or non-morbid varieties of the cervix and os uteri. A large uterus, especially at its lower part, a large and soft cervix, a patulous os, fissured, indurated, and cicatrized, may all exist, Dr. Ashwell says, without organic, and especially active organic disease. To the latter part of this proposition we assent, but do not feel so satisfied with the correctness of the former.

The remarks upon the speculum are short, and contain nothing new: the author has selected a favorite form of instrument, and of course likes it best. It is a conical tube, made of tin, with a highly polished inner surface: he uses a series of these of various sizes, and judges of the size best adapted to the individual case from the previous introduction of the finger. The stethoscope, as an aid to diagnosis, is dealt with very concisely. He seems afraid of reposing too much confidence on the "placental souffle," considering it liable to be confounded with a *bruit* in a large artery, the result of pressure, and *vice versâ;* and yet in the two cases he adduces, one of pregnancy, and one of hard tumour of the uterus, he states that this sign (that is, the presence of this "bruit,") did not embarrass the diagnosis: the bruit ceasing with the altered position of the tumour.

Dr. Ashwell objects to a diagnosis derived from an investigation of the discharges simply, and with justice; but, as he purposes again treating of this subject, we shall reserve any remarks we might feel disposed to make for a future opportunity.

### VI. *Observations on Abdominal Tumours and Intumescence.* By Dr. Bright.

This is a most admirable paper; but, as it is only the first of a series of the same kind, we shall postpone the analysis of it to a future occasion. The following is the general outline, given by Dr. Bright, of the various subjects which he purposes to comprehend in his survey of this most interesting field of pathological and practical enquiry. It is gratifying to know that the powers of the enquirer are commensurate with the difficulties of the subject of investigation.

"1. The Integuments; including various cutaneous changes, polysarcia—anasarca—malignant deposits—abscesses—protrusions.

"2. The Peritoneum—the various results of inflammatory action; as effusion, including ascites—adhesions, and various depositions of adhesive matter—tubercular deposits—malignant diseases—hydatids.

"3. The Stomach; including flatulent distention—chronic disease—malignant changes.

"4. The Intestines; including flatulent distentions—retained fæces, and other matters—mechanical obstruction—malignant strictures.

"5. The Liver; enlarged from congestion—forced down by the lungs or by effusion—distended with bile—enlarged by various changes of the structure generally—enlarged by malignant growth.

"6. The Spleen; enlarged by congestion—changed in structure.

"7. The Pancreas; enlarged, or hardened.

"8. The Mesenteric Glands; simple enlargement—scrofulous, malignant, and osseous changes.

"9. The Kidneys; enlarged by vesicles, by abscess, by malignant disease—distended ureter.

"10. The Bladder; distended—thickened—forced forward.

"11. Uterus; enlarged from pregnancy—chronic increase—scirrhous disease, and other changes.

"12. Ovaries; enlarged by simple cysts—by malignant growths.

"13. Extra-uterine Fœtation.

"14. Aneurismal Tumours; cæliac—aortal—iliac." (p. 433.)

### VII. *On the Influence of Electricity as a Remedy in certain Convulsive and Spasmodic Diseases.* By Dr. Addison.

Dr. Addison confesses that he formerly attached as little value to electricity as a remedial agent as is ascribed to it by the profession in general, being "led greatly to underrate its efficacy, either in consequence of its vague and indiscriminate recommendation, or from the inefficient and careless manner in which it had been applied." The convulsive and spasmodic disorders of females, in which, under a happier direction of its use, he has, with the assistance of Mr. Golding Bird, recently and successfully employed it, were, for the most part, such as are connected with some irregularity in the menstrual discharge.

The mode in which electricity was employed was either by taking sparks along the course of the spine, or in the form of shocks passed through the pelvis. In the former case, the patient being insulated, the sparks were taken in rapid succession, at the distance of about an inch, for about five to ten minutes, until an eruption followed, which closely resembled Lichen urticarius. In the latter case, a large-sized Leyden jar was discharged through the pubes and sacrum, the intensity of the charge being regulated by the electrometer. In one case only, when the patient's strength was not sufficient to admit of the ordinary mode, the magnetic-electrical machine was substituted. The seven cases detailed are, as we are informed, not selected ones, but all those indiscriminately in which he has lately tested this remedy; so that they may be considered as affording a fair estimate of its value in such affections. In the first case, (the only one which we have space to enter into,) a girl of seventeen, well developed, but rather of a nervous temperament, who had been from the age of fourteen the subject of painful menstruation recurring every fortnight, on being frightened during one of her periods, experienced a sudden interruption of the evacuation, which was followed by hysterical fits, with continual trembling of the limbs. Pain in the head, back, and loins supervened, along with palpitations. Her mouth became liable to frequent distortions, and the limbs, the upper ones especially, were violently agitated; the hands revolving round each other at regular intervals, in a very systematic manner. These symptoms, after having been long ineffectually combated by a host of remedies, including bleeding, blistering, purgatives, creosote, prussic acid, sulphate of zinc, carbonate of iron, cold affusion, &c., yielded, in a great degree, to the sulphate of iron in half-drachm doses, aided by a combination of calomel, colocynth, aloes, &c., and the use of the shower-bath. Two months after leaving the hospital convalescent for the sea-side, she returned in a worse state than ever; epileptic paroxysms, accompanied with episthotonos during the fit, having taken the place of the chorea. The legs appeared paralytic, and the amenorrhœa persisted. The same tonic and purgative remedies which had formerly been used with success were again employed, but now without benefit. Electro-magnetism was had recourse to; and, after three weeks of its employment, her general health was much improved: she could now hold her needle, and the fits had become slighter, though not yet less frequent. Electrical sparks were now drawn from the spine on alternate days, each exhibition inducing a vivid eruption. In a week she was able to walk across the room unassisted; her countenance was less anxious, and the fits less frequent. Twelve shocks through the pelvis, every second day, were next substituted: their very first employment was followed by severe abdominal and pelvic pain, and by the reappearance of the menses. In the following month, the uterine evacuation was recalled by a repetition of the same means; soon after which she left the hospital entirely free from chorea, though still subject to fits of diminished force and frequency: a result which, though not altogether satisfactory, proves at least that the agent employed was one of no inconsiderable efficacy.

In the second case, that of a girl of fourteen, for years afflicted with chorea, a complete cure was effected by taking sparks from the spine for the space of three weeks. The third case was a similar one, in a boy of the like age, and the result equally favorable, and as rapidly attained. In the fourth, a girl of sixteen, the menses being suddenly interrupted by cold, an hysterical attack supervened, accompanied by a sense of numbness and coldness in the left side of the body. The left eye soon afterwards became amaurotic, and the lid dropped; there was defect of muscular power over the whole left side, along with headach and giddiness. After cupping, blistering, and aperients had failed, electricity, in the form of sparks, was ordered. The power over the eyelid and limbs was soon regained, and the menstrual function reestablished. The amaurosis, however, was permanent. The fifth case, which somewhat resembled the first, though much milder, had been considerably benefited by electricity, but was still under treatment. In the sixth and seventh cases, both likewise instances of chorea, electricity was quite successful.

The above cases are certainly of a nature in some degree to revive our expectations from electricity employed as a remedial agent, when used in appropriate circumstances, and with sufficient energy and perseverance. We trust it will lead others who have extensive hospital opportunities to test it still more fully in cases of the kind just described. The period which seemed necessary to the production of its full effect varied from three to six weeks. For fuller details as to the precise mode of its exhibition, we refer to the paper itself.

The remaining papers we are compelled to pass over with little more than a mere notice of the subjects treated of.

VIII. *Case of Disease in the Fœtus.* By Mr. T. W. King.

IX. *Description of the Sacculus or Pouch in the Human Larynx.* By Mr. John Hilton.

X. Under the head of "*Ophthalmic Cases occurring at the Guy's Hospital Eye Infirmary*," we have two instances of imperfect amaurosis (chronic retinitis), treated by Mr. Morgan, in which inunction of the temples, night and morning, with the ointment of veratria, coupled, in the first case, with fractional doses of the extract of the same plant taken internally, appeared to be useful: but, as this remedy was used in combination, in one or other of these cases, with such powerful auxiliaries as cupping, mercurials, and continued purgatives, it would require much more extensive and unequivocal premises to enable the medical public to decide on the efficacy of veratria in amaurotic disease. We think, however, evidence sufficient has been already brought forward to justify, if not very strong hopes as to its utility, at least further and fuller trials of this powerful agent. Besides the gradual improvement of vision, it is stated to cause a sensation of pricking in the temples, and an increased frequency of the flashes of light.

XI. *Experiments and Observations on Albuminous Fluids.* By Dr. Babington.

In this paper the changes which the fluids in question undergo by a union with the pure alkalies, and also with the neutral salts, are investigated. On the addition of a strong solution of pure potass, for example, to pus or to white of egg, a transparent, tenacious, gelatinous mass is formed, which is very little soluble in water, and is not precipitated by dilute nitric acid. With serum, also, a thick tenacious fluid is the result; approaching less, however, to the solid condition than in the preceding instances. Dr. B. considers that we may turn the above facts to account in practice; as, for instance, in those cases where we are in doubt as to a urinary deposit, whether it be purulent or not. In the albuminous urine of renal disease, however, he has hitherto in vain endeavoured to produce the above appearance.

It was long ago stated by John Hunter that pus is coagulated by sal ammoniac: but, in the possession of this power, the hydrochlorate of ammonia does not stand alone; for Dr. B. finds that the hydrochlorate of soda, nitrate of potass, the sulphates of magnesia, of soda, or of potass, all equally form viscid compounds with pus. Dr. B. conceives it probable that natural mucus is formed by some combination analogous to that which results from the action of a pure alkali or neutral salt on pus or albumen. But, as Dr. B.'s experiments and conclusions have been in a great degree anticipated by others, (a fact of which he became aware only just before publication,) we do not think it necessary to enter further into them here.

Since this article was sent to the press, we have received the Sixth Number of the "Reports," the notice of which we must postpone to a future opportunity. We are delighted to perceive that the medical officers of Guy's Hospital bid fair to falsify our prophecy in a former Number, that the supply of materials worthy of the high reputation of the "Reports" cannot be long expected to continue. The Number just published seems worthy of its predecessors, and of the distinguished institution whence it emanates.

---

